# An Interaction of Renin-Angiotensin and Kallikrein-Kinin Systems Contributes to Vascular Hypertrophy in Angiotensin II-Induced Hypertension: *In Vivo* and *In Vitro* Studies

**DOI:** 10.1371/journal.pone.0111117

**Published:** 2014-11-04

**Authors:** Graziela S. Ceravolo, Augusto C. Montezano, Maria T. Jordão, Eliana H. Akamine, Tiago J. Costa, Ana P. Takano, Denise C. Fernandes, Maria L. Barreto-Chaves, Francisco R. Laurindo, Rita C. Tostes, Zuleica B. Fortes, Renato P. Chopard, Rhian M. Touyz, Maria Helena C. Carvalho

**Affiliations:** 1 Department of Pharmacology, Institute of Biomedical Sciences, University of Sao Paulo, Sao Paulo, Brazil; 2 Department of Physiological Sciences, Biological Sciences Center, State University of Londrina, Londrina, Brazil; 3 Ottawa Health Research Institute, Kidney Research Centre, University of Ottawa, Ottawa, Canada; 4 BHF Glasgow Cardiovascular Research Centre, University of Glasgow, Glasgow, United Kingdom; 5 Department of Anatomy, Institute of Biomedical Sciences III, University of São Paulo, Sao Paulo, Brazil; 6 Department of Pharmacology, Ribeirão Preto Medical School, University of Sao Paulo, Sao Paulo, Brazil; 7 Vascular Biology Laboratory, Heart Institute, University of Sao Paulo, Sao Paulo, Brazil; Albany Medical College, United States of America

## Abstract

The kallikrein-kinin and renin-angiotensin systems interact at multiple levels. In the present study, we tested the hypothesis that the B1 kinin receptor (B1R) contributes to vascular hypertrophy in angiotensin II (ANG II)–induced hypertension, through a mechanism involving reactive oxygen species (ROS) generation and extracellular signal-regulated kinase (ERK1/2) activation. Male Wistar rats were infused with vehicle (control rats), 400 ng/Kg/min ANG II (ANG II rats) or 400 ng/Kg/min ANG II plus B1 receptor antagonist, 350 ng/Kg/min des-Arg^9^-Leu^8^-bradykinin (ANGII+DAL rats), via osmotic mini-pumps (14 days) or received ANG II plus losartan (10 mg/Kg, 14 days, gavage - ANG II+LOS rats). After 14 days, ANG II rats exhibited increased systolic arterial pressure [(mmHg) 184±5.9 vs 115±2.3], aortic hypertrophy; increased ROS generation [2-hydroxyethidium/dihydroethidium (EOH/DHE): 21.8±2.7 vs 6.0±1.8] and ERK1/2 phosphorylation (% of control: 218.3±29.4 vs 100±0.25]. B1R expression was increased in aortas from ANG II and ANG II+DAL rats than in aortas from the ANG II+LOS and control groups. B1R antagonism reduced aorta hypertrophy, prevented ROS generation (EOH/DHE: 9.17±3.1) and ERK1/2 phosphorylation (137±20.7%) in ANG II rats. Cultured aortic vascular smooth muscle cells (VSMC) stimulated with low concentrations (0.1 nM) of ANG II plus B1R agonist exhibited increased ROS generation, ERK1/2 phosphorylation, proliferating-cell nuclear antigen expression and [H3]leucine incorporation. At this concentration, neither ANG II nor the B1R agonist produced any effects when tested individually. The ANG II/B1R agonist synergism was inhibited by losartan (AT1 blocker, 10 µM), B1R antagonist (10 µM) and Tiron (superoxide anion scavenger, 10 mM). These data suggest that B1R activation contributes to ANG II-induced aortic hypertrophy. This is associated with activation of redox-regulated ERK1/2 pathway that controls aortic smooth muscle cells growth. Our findings highlight an important cross-talk between the DABK and ANG II in the vascular system and contribute to a better understanding of the mechanisms involved in vascular remodeling in hypertension.

## Introduction

The kallikrein-kinin (KKS) and renin-angiotensin (RAS) systems play a key role in multiple physiological and pathophysiological conditions, including blood pressure regulation, vascular smooth muscle cells (VSMC) growth, and inflammation. The KKS and RAS systems also interact at multiple levels, therefore, changes in the activity of one system greatly impact the activity of the other [1].

Angiotensin II (ANG II) is the main RAS vasoactive peptide. The cellular effects of ANG II are mediated by at least two receptors subtypes, AT1 and AT2, which belong to the seven-transmembrane G protein-coupled receptor (GPCR) superfamily [2]. ANG II through AT1 receptor plays a key role in blood pressure homeostasis and VSMC proliferation [3].

Kinin B1 (B1R) and B2 (B2R) receptors are GPCRs, which mediate kinins effects. B2R is expressed constitutively and induces the classical effects of the nonapeptide hormone bradykinin, which is one of the KKS effectors [4]. B1R mediates the actions of des-Arg^9^-bradykinin (DABK), a metabolite of bradykinin. B1R is weakly expressed in healthy tissues, but its expression is enhanced during tissue injury, by proinflammatory cytokines or by growth factors [4].

Originally described as an important regulator of inflammatory processes [5], the function of B1R upregulated in the cardiovascular system is not completely understood. It has been described that B1R contributes to the protective effect of angiotensin converting enzyme inhibitors in mice after myocardial infarction [6]. On the other hand, the B1R upregulation has also been associated with hypertension [7] and the development of vascular diseases, such as atherosclerosis [8], [9].

VSMC growth is a prominent feature of the vascular disease process and it is associated with activation of a number of signaling molecules, including mitogen-activated protein kinase (MAPK) [10]. Intriguingly, DABK, B1R agonist, potentially stimulates MAPK activity in cultured VSMC [7], and it is possible that one of the vascular functions of B1R is to induce VSMC growth [9].

Hypertension is an important and potent risk factor for the development of vascular disease. We demonstrated, in different models of hypertension, that B1R expression is increased in the vascular tissue of hypertensive animals [11], [12]. This positive modulation of B1R expression is dependent on ANG II/AT1 receptor, involves reactive oxygen species (ROS) generation and nuclear translocation of nuclear factor kappa-B (NF-κB) [11], [12]. However, the role of B1R in vascular hypertrophy in hypertension is not clear. Therefore, we determined the functional role of B1R in vascular hypertrophy associated with ANG II-dependent hypertension. We also sought to understand the molecular mechanisms underlying the crosstalk between ANG II and B1R activation in VSMC, focusing on signaling events involving ROS generation and MAPK activation.

## Materials and Methods

### Animals

Experiments were performed in male Wistar rats (n  = 36) weighing 180–200 g, obtained from the breeding stock of the Institute of Biomedical Sciences of the University of Sao Paulo (ICB-USP). Rats were kept in a temperature-controlled room on a 12-hour light/dark cycle, 60% humidity, standard rat chow and water *ad libitum*. All the procedures used in this study were approved and performed in accordance with the guidelines of the Animal Ethics Committee of the ICB-USP (Permit Number: 145, page 95, book 02), following the Guidelines for the Care and Use of Laboratory Animals published by the US National Institutes of Health (NIH Publication No. 85-23, revised 1996).

### Induction of hypertension, B1R and AT1 antagonism

The induction of hypertension with ANG II was performed as previously described [12]. Briefly, rats were anesthetized with xylazine (7.4 mg/Kg, ip) and ketamine (113 mg/Kg, ip) and osmotic minipumps (Alzet 2002) containing either ANG II 400 ng/Kg/min (ANG II group, n = 11) or saline 0.9% (control group, n = 11) were subcutaneously implanted in the animals. In another group, the des-Arg^9^-Leu^8^-bradykinin, a selective B1R antagonist [13], [14], was simultaneously infused with ANG II (ANG II + DAL group, n = 10). The B1R antagonist was infused subcutaneously by osmotic minipumps at a rate of 350 ng/Kg/min. All infusions were performed for 14 days [15]. In another series of experiments, ANG II infused-rats, were simultaneously treated with losartan (10 mg/kg by gavage for 14 days; ANG II+LOS; n = 5). Systolic arterial pressure was measured in conscious rats by tail-cuff plethysmography (PowerLab 4/S, AD Instruments Pty Ltd) at zero, 7, and 14 days after the minipump implantation, as previously described [12] and calculated as the average of three consecutive measurements.

### Real-time PCR

The mRNA expression of B1 and AT1 receptors was determined by real-time PCR, as previously described [11]. On the 14^th^ day after minipump implantation, the rats were anesthetized with xylazine (7.4 mg/Kg, ip) and ketamine (113 mg/Kg, ip), the thorax was opened and the descending aorta was excised. Total cellular RNA was isolated from the aorta using Trizol Reagent. Total RNA (2 µg) was used for first-strand cDNA synthesis using SuperScript II. cDNA samples were submitted to real-time PCR amplification using Platinum SYBR QPCR Supermix-UDG and specific oligonucleotides (Invitrogen Co., San Diego, CA) for: B1R (forward-GCATCCCCACATTCC TTC TA; reverse-AAGAAGTGGTAAGGGCACCA); AT1 receptor (forward-CACTTTCCTGGATGTGCTGA; reverse-CCCAGAAAGCCGTAGAACAG), and β-actin (forward-AAGATTTGGCACCACACTTTCTACA; reverse-CGGTGAGCAGCACAGGGT) that was used as an internal control. Real-time PCR reactions were performed, recorded, and analyzed using the Corbett Research system (Corbett Life Sciences, Sydney, Australia). Expression data were calculated from the cycle threshold (Ct) value using the ΔΔCt method for quantification. The expression of β-actin mRNA was used for normalization and values expressed as fold of control.

### Morphological analysis

For the analysis of vascular hypertrophy, animals were perfused in fixed pressure with 4% sodium phosphate-buffered paraformaldehyde (4% PFA). The aorta was then removed and fixed in 4% PFA, embedded in paraffin and cut into transversal sections (4 µm). Samples were stained with hematoxylin-eosin. In the morphological analysis, the external (EL), internal (IL) elastic lamina and external (ED) and internal (ID) diameters were determined and the cross-sectional area (CSA = EL-IL), wall thickness (δ = ED-ID/2) and the ratio media/lumen (media/lumen = δ/Di) were calculated. Digital photomicrographs of aortic sections were analyzed using KS300 software, with the wall thickness defined as the distance between the outer and inner elastic lamine. A single investigator unaware of the experimental groups performed the analysis.

### ROS detection in aorta

The ROS generation in aorta was evaluated by HPLC analysis and transverse sections fluorescence analysis, both involving dihydroethidine (DHE) oxidantion. The DHE oxidation yields at least two fluorescent products, 2-hydroxyethidium (EOH), known to be more specific for superoxide anion, and the less-specific product ethidium (E). Both dihydroethidine fluorescent products bind to DNA and can be detected by virtue of its red fluorescence [16]. The analysis of ROS by HPLC was performed as described previously [16], [12]. Briefly, aorta segments, ≈10 mm in length, were incubated in DHE (50 µmol/L) in the dark (37°C, 30 min). Aortic segments were homogenized in liquid nitrogen with pestle, suspended in acetonitrile, centrifuged, and the supernatants injected into a high-performance liquid chromatography system. DHE and DHE-derived products, such as 2-hydroxyethidium (EOH) and ethidium derived from oxidation of DHE by superoxide anion and by other reactive oxygen species, were determined using ultraviolet and fluorescence detection, respectively. Thus, the DHE-derived products were expressed as a ratio of EOH and E generation per DHE consumed (initial DHE concentration minus remaining DHE). To determine ROS generation in transverse aortic sections, aortic segments were embedded in tissue freezing medium and snap-frozen. 10 µm thick cryosections were incubated in a light-protected and humidified chamber (37°C, 30 min) with DHE 5 µM. Fluorescent images were detected with a 585–590 nm long-pass filter, under a microscope (Axioskop, Zeiss) with a x40 objective lens coupled to a digital camera.

### Aortic smooth muscle cell primary culture

In order to investigate the possible mechanism responsible for the contribution of B1R activation in ANG II signaling, aortic VSMC from Wistar rats were examined. The study was approved by the Animal Ethics Committee of the University of Ottawa and performed according to recommendations of the Canadian Council for Animal Care. Adult male Wistar rats were killed by decapitation. VSMC derived from aorta were isolated and characterized as described in detail previously [17]. In brief, aorta was cleaned of adipose and connective tissue, and VSMC were dissociated by digestion of vascular arcades with enzymatic solution (collagenase, elastase, soybean trypsin inhibitor and BSA type I; 60 min, 37°C). The tissue was filtered and the cell suspension centrifuged and re-suspended in Dulbecco Minimal Essential Medium (DMEM) containing 10% fetal calf serum, 2 mM glutamine, 20 mM Hepes (pH 7.4) and antibiotics. Each cultured cell line was initially prepared from aorta pooled from 10 rats. Cells were cultured until passage 3 before being frozen for storage in liquid nitrogen, and once thawed did not undergo repeated freeze-thaw cycles. Cells were cultured in DMEM, supplemented with 10% fetal bovine serum. At least four different primary cell cultures were used between passages 4 and 7 [17]. For the experiments involving ANG II and B1R agonist interaction the culture medium was replaced with serum-free medium for 24 h to render the cells quiescent.

To characterize the effect of ANG II in B1R expression, cells were stimulated with ANG II (100 nM) for 0, 2, 4, 6, 8, 10 and 24 h. The synergistic effect of ANG II and the B1R agonist DABK was evaluated by stimulating cells either with ANG II and/or DABK, at low (0.1 nM) and high (100 nM) concentrations, for 5 minutes. Low concentrations were those that alone did not produce any effect on ERK1/2 phosphorylation and high concentrations were those that individually induced nearly maximal kinase phosphorylation [17], [18]. The low and high concentrations were determined by performing concentration-effect curves (data not shown) and by previous studies from our group [18]. This protocol was also performed in cells preexposed for 30 minutes to: 10 µM losartan (LOS - selective AT1 receptor antagonist), 10 µM des-Arg^9^-Leu^8^-bradykinin (DAL - selective B1R antagonist) or 1 mM Tiron (superoxide anion scavenger).

### Protein analysis by Western blot

Proteins were extracted from aorta and VSMC in culture, separated by electrophoresis on a 10% polyacrylamide gel, and transferred onto a nitrocellulose membrane, as previously described [18]. Nonspecific binding sites were blocked with 5% skim milk in Tris-buffered saline solution with Tween for 1 h at 24°C. Membranes were then incubated with phospho-specific antibodies (1∶1000) overnight at 4°C. Antibodies were incubated with anti-extracellular signal-regulated kinase (ERK1/2) (Thr202/Tyr204 - Cell Signaling). The ERK1/2 nonphosphoantibodies (1∶2000) were also used in the present study (Cell Signaling). In other series of experiments membranes from VSMC were then incubated with anti-B1R (1∶1000) (Santa Cruz Biotechnology, Santa Cruz, California, USA) or anti-proliferating-cell nuclear antigen (PCNA) (Cell Signaling) and also anti-β-actin (Santa Cruz Biotechnology, Santa Cruz, California, USA) at 4°C. β-actin was used as a housekeeping protein. After incubation with secondary antibodies, signals were revealed with chemiluminescence, visualized by autoradiography, and quantified densitometrically. Results were normalized by the total protein and expressed as percentage of vehicle used in the experimental protocols for cells and control aorta.

### VSMC protein synthesis

The protein synthesis was quantified on the basis of tritiated leucine incorporation [19]. VSMC were stimulated with ANG II (0.1 nM) and the B1R agonist, DABK (0.1 nM) alone or in association, for 24 hours. The same protocol was performed after pre-exposing cells for 30 min to: 10 µM LOS and 10 µM DAL. The VSMC were also stimulated with ANG II in high concentration (100 nM) for 24. At 6 h before harvest, L-[3,4,5-3H] leucine (5 µCi/ml) was added to the culture medium to measure incorporation into newly synthesized protein. Total cellular proteins were precipitated in ice-cold 10% trichloroacetic acid and collected by centrifugation (14,000 g for 10 min at 4°C). The proteins pellets were washed twice by re-suspension in cold 10% trichloroacetic acid and collected by centrifugation. The final pellets were dissolved in 0.2 N NaOH by incubation at 60°C for 30 min. Radioactivity was measured by liquid scintillation counting. Protein concentrations were determined with protein assay reagent (Bio-Rad Laboratories). The [H3] leucine incorporation was normalized by the total protein and demonstrated as percentage of control cells (vehicle).

### Detection of superoxide anion in VSMC by lucigenin chemiluminescence

The lucigenin-derived chemiluminescence assay was used to determine superoxide anion generation [20]. VSMC were stimulated with ANG II (0.1 nM) and the B1R agonist DABK (0.1 nM) alone or in association, for 5 min. The same protocol was performed after pre-exposing cells for 30 min to: 10 µM LOS, 10 µM DAL or 1 mM Tiron. In another series of experiments, VSMC were stimulated with ANG II in high concentration (100 nM), for 5 min, after being pre-exposed for 30 min to: vehicle, 10 µM LOS or 10 µM DAL. Superoxide anion production was measured by lucigenin-enhanced chemiluminescence. The reaction was started by the addition of NADPH (0.1 mM) to the suspension (250 µl of final volume) containing sample (50 µl), lucigenin (5 µM) and assay buffer [50 mM of KH_2_PO_4_, 1 mM of EGTA, and 150 mM of sucrose (pH 7.4)]. Luminescence was measured every 1.8 s for 3 min in a luminometer (Orion Luminometer, Berthold detection systems). Buffer blank was subtracted from each reading. Superoxide anion production was expressed as the percentage increase from baseline values. Protein concentrations were determined with protein assay reagent (Bio-Rad Laboratories).

### Drugs and reagents

Angiotensin II and des-Arg^9^-Leu^8^-bradykinin were purchased from Bachem Bioscience Inc. (Pensylvania, USA). Antibodies were purchased from Santa Cruz Biotechnology or Cell Signaling Technology (Beverly, USA).

### Statistical analysis

Data are means ± SEM of *n* samples. B1R and AT1 mRNA levels were measured relative to β-actin mRNA levels. B1R and PCNA protein levels were measured relative to β-actin protein. p-ERK1/2 protein expression was normalized to total ERK1/2 levels. Statistical analysis was performed with GraphPad Prism software. Results were analyzed by one-way ANOVA in conjunction with a Bonferroni post-test for in vivo study or by one-way ANOVA followed by Dunnett's test for multiple comparisons with control cells (vehicle). *P*<0.05 was considered significant.

## Results

### ANG II increases B1R expression in aorta and in VSMC

**Figure 1:**
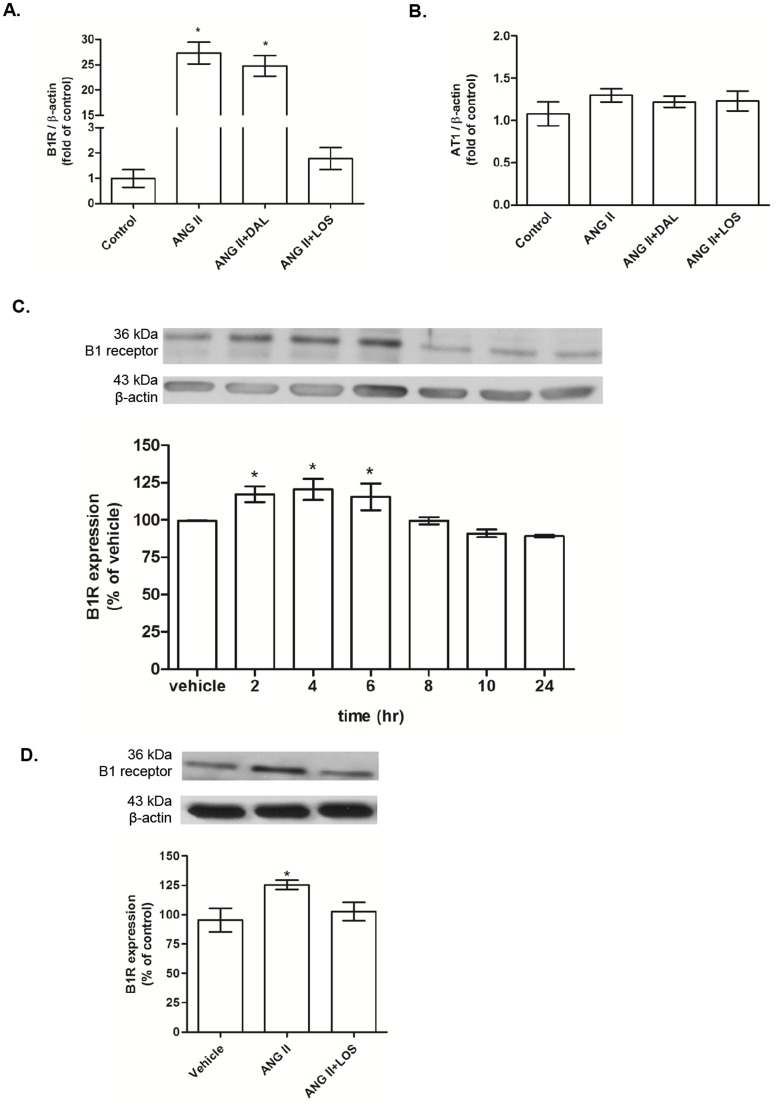
B1 and AT1 receptors expression in aorta and aortic smooth muscle cells (VSMC) primary culture. Bar graph shows B1 receptor (B1R) [A] and AT1 receptor [B] mRNA levels in aortas from control, ANG II (400 ng/Kg/min), ANG II (400 ng/Kg/min) + DAL (350 ng/Kg/min) and ANG II (400 ng/Kg/min) + LOS (10 mg/Kg/day) rats. The receptors mRNA expression was calculated from the cycle threshold (Ct) value using the Δ^2^Ct method for quantification. Values were normalized against β-actin mRNA. Data represented mean±SEM; n = 5–7 for each group; *P<0.05 vs control. [C] Bar graph shows the temporal effects of ANG II (100 nM, 0–24 h) on protein B1R expression in aortic VSMC. [D] Bar graph shows B1R expression in aortic VSMC after stimulation with ANG II (100 nM, 2 h), in the presence or not of AT1 antagonist, losartan (LOS – 10 µM). B1R expression was normalized against the housekeeping protein β-actin. Data are presented as mean±SEM of 4 experiments. *P<0.05 vs. control.

ANG II infusion (400 ng/Kg/min) increased aortic B1R mRNA expression in comparison with control. The B1R antagonist did not interfere with the effects of ANG II on B1R expression (Figure 1A). Treatment with LOS inhibited ANG II-induced increased aortic B1R mRNA expression. Neither ANG II, ANG II+DAL nor ANG II+LOS infusion altered AT1 receptor mRNA vascular expression when compared to those in aortas of control rats (Figure 1B). We also examined the effect of ANG II on B1R protein expression in aortic VSMC at different time points. As shown in Figure 1C, ANG II (100 nM) increased B1R expression in a time-dependent manner, with a maximal increase at 2 h that last for up to 6 h when compared to unstimulated cells. B1R protein expression returned to basal levels at 8h post-ANG II stimulation and remained up to 24 h. LOS incubation blunted ANG II effects on B1R expression (Figure 1D).

### Effect of B1R and AT1 antagonism on systolic arterial pressure levels and aortic morphology

Table 1 summarizes the data on systolic arterial pressure levels and aortic wall morphology. ANG II infusion for 14 days resulted in a progressive increase in systolic arterial pressure in comparison with the control group, measured at the 7^th^ and 14^th^ days. Treatment with the B1R antagonist did not interfere with the increase in systolic arterial pressure observed in ANG II-infused rats. On the other hand, the AT1 receptor antagonist LOS blunted ANG II-induced hypertension.

**Table 1:** Temporal effect of angiotensin II (ANG II) infusion alone, or associated with des-Arg^9^-Leu^8^-bradykinin (DAL), or losartan (LOS) on systolic arterial pressure and the effect of ANG II, ANG II+DAL and ANG II+LOS, 14 days of infusion on aorta morphology.

	Control	ANG II	ANG II+DAL	ANG II+LOS
***Systolic arterial pressure (mmHg)***				
Days of infusion				
0	116±1.9 (11)	116±1.5 (11)	119±1.5 (10)	117±2.4 (5)
7	115±2.1 (11)	148±2.6* (11)	150±6.6* (10)	124±8.2** (5)
14	115±2.3 (11)	184±5.9* (11)	187±8.7* (10)	125±4.3** (5)
***Aorta***				
Wall thickness (µm)	101±7.1 (7)	132±9.1* (7)	114±8.5 (7)	108±6.7** (5)
CSA (µm^2^.10^−3^)	451±19 (7)	640±36* (7)	556±63 (7)	487±57** (5)
W/L (10^−2^)	6.5±0.2 (7)	7.6±0.4 (7)	6.6±0.4 (7)	6.5±0.4(5)

Systolic arterial pressure and thoracic aorta wall morphology. Systolic arterial pressure temporal evaluation in ANG II (400 ng/Kg/min), ANG II (400 ng/Kg/min)+DAL (350 ng/Kg/min) and ANG II (400 ng/Kg/min)+LOS (10 mg/Kg/day, gavage) rats. Thoracic aorta wall morphology in control, ANG II (400 ng/Kg/min), ANG II (400 ng/Kg/min)+DAL (350 ng/Kg/min) and ANG II (400 ng/Kg/min)+LOS (10 mg/Kg/day, gavage) rats. Aorta cross-sectional area (CSA), wall thickness and wall/lumen (W/L) ratio. Data are represented as mean±SEM; (n) indicates the number of animals in each group *P<0.05 vs control; **P<0.05 vs ANG II.

After 2-weeks of ANG II infusion (400 ng/Kg/min) the CSA and wall thickness were increased approximately 40% and 31%, respectively, when compared with control. B1R antagonism reduced and AT1 receptor antagonism prevented the ANG II-induced changes in CSA and wall thickness. There were no differences in W/L ratio among the groups.

### B1R antagonism reduces vascular ROS generation and ERK1/2 phosphorylation in ANG II-infused rats

**Figure 2:**
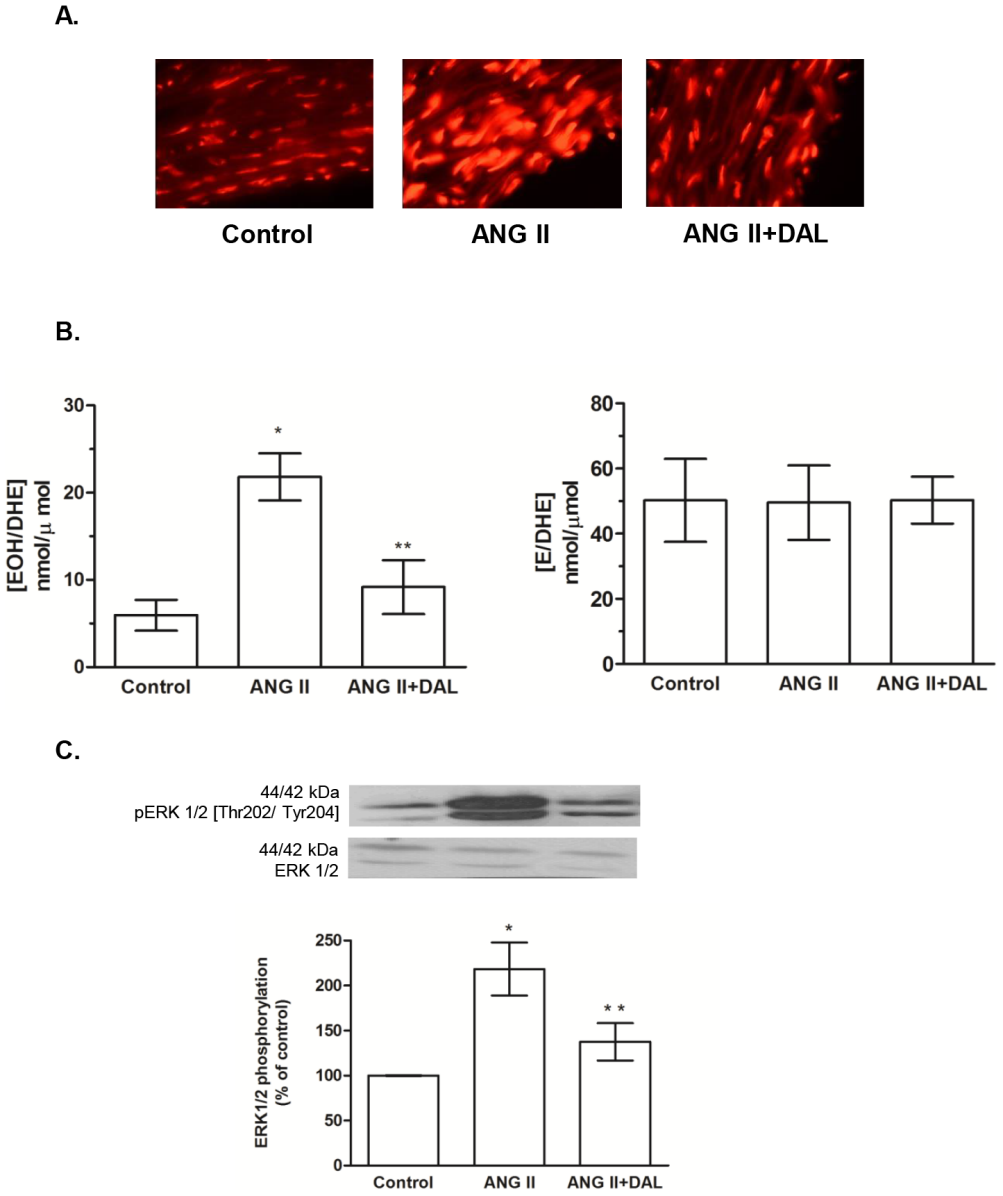
B1 receptor antagonism prevented angiotensin II (ANG II) effect in aorta. [A] Fluorescence microscopy of aortic transverse sections after incubation with DHE. [B] Ratios of 2-hydroxyethidium/dihydroethidine (EOH/DHE) and ethidium/dihydroethidine (E/DHE) obtained by HPLC analysis from aortic segments. Data are expressed as mean ± SEM of 5 rats for each group. *P<0.05 vs control and **P<0.05 vs. ANG II [C] Bar graph shows the ratio of phosphorylated/total ERK1/2 that was used as an indicator of ERK1/2 activity. Data are expressed as mean ± SEM of 6 rats for each group. *P<0.05 vs control and **P<0.05 vs. ANG II.

Aortic sections from ANG II-infused rats exhibited increased ROS generation, demonstrated as enhancement of the red fluorescent dots when compared with control rat aortas. Treatment with the B1R antagonist reduced ROS generation in aortas from ANG II-infused rats (Figure 2A). The HPLC analyses demonstrated a higher EOH/DHE concentration in aortas of the ANG II rats when compared with the control rats, and the B1R antagonist treatment corrected that. In contrast, no alteration in E/DHE generation has been observed among the groups (Figure 2B). ERK1/2 phosphorylation was increased in aortas from ANG II-infused rats when compared with control and ANG II+DAL rat aortas (Figure 2C).Those data suggest that B1R activation contributes to ANG II-AT1 signaling in the vascular tissue.

### ANG II and B1R agonist have synergistic effects on NADPH oxidase-induced superoxide anion generation in aortic VSMC

**Figure 3:**
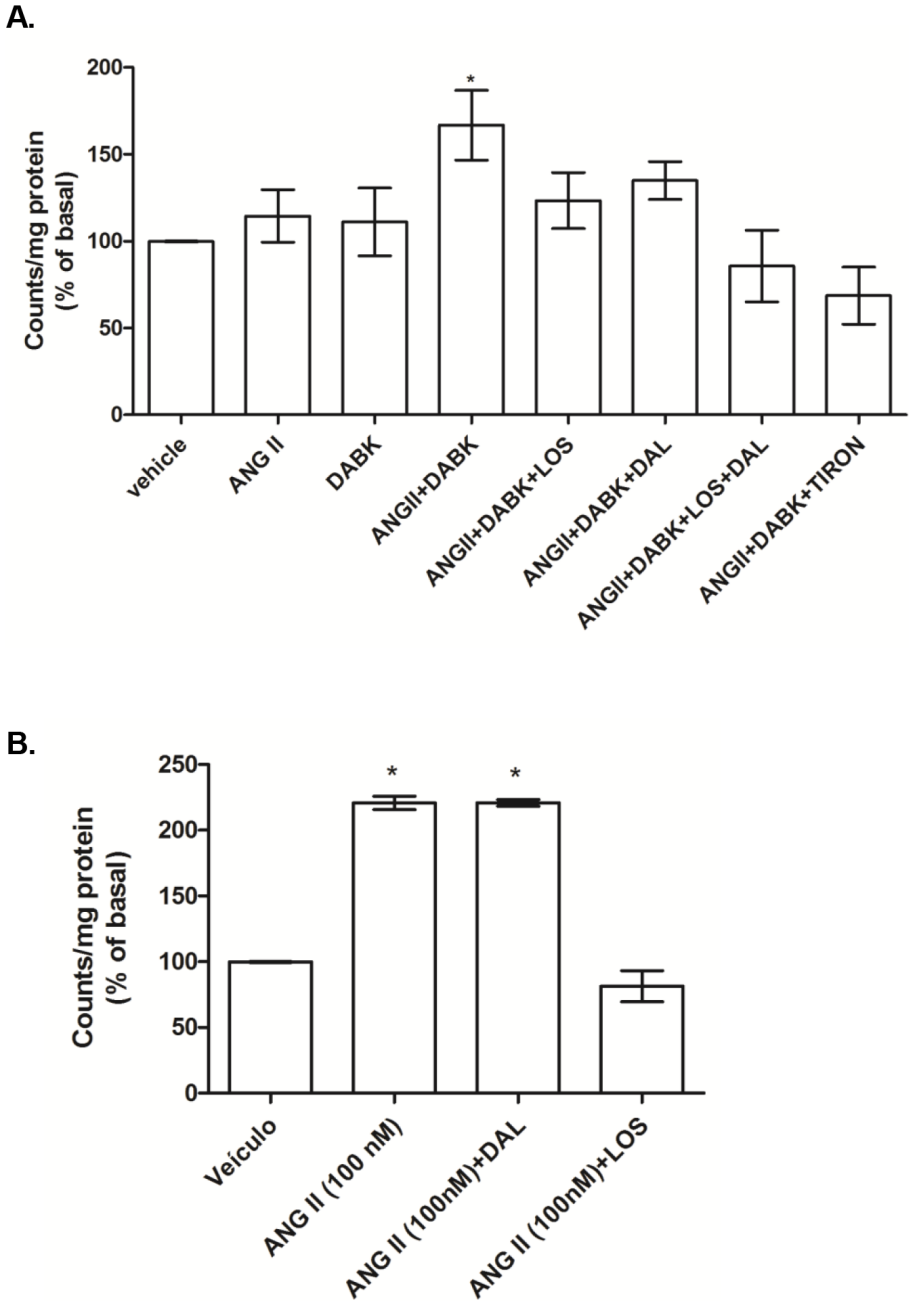
Effect of angiotensin II (ANG II) and des-Arg^9^-bradykinin (DABK) on superoxide anion generation in VSMC. [A] Bar graph demonstrates the effects of ANG II (0.1 nM), DABK (0.1 nM) and ANG II-DABK co-stimulation for 5 min, in the presence of either vehicle, AT1 antagonist losartan (LOS – 10 µM), B1 receptor antagonist des-Arg^9^-Leu^8^-bradykinin (DAL – 10 µM) or superoxide anion scavenger (TIRON - 1 mM) on superoxide anion generation. Results were expressed as mean±SEM of 5 experiments. *P<0.05 vs vehicle. [B] Bar graph demonstrates the effects of ANG II (100 nM) stimulation for 5 min, in the presence of vehicle, AT1 antagonist, losartan (LOS – 10 µM), or B1 receptor antagonist, des-Arg^9^-Leu^8^-bradykinin (DAL – 10 µM). Results are represented as mean ± SEM of 5 experiments. *P<0.05 vs vehicle.

In order to determine if there is a synergistic effect between AT1 and B1 receptors signaling, primary cultures of aortic VSMC were treated with ANG II and DABK in a low (0.1 nM) concentration. At this concentration, neither ANG II or DABK alone induced NADPH oxidase activation, but when applied together a significant increase of NADPH oxidase-derived superoxide anion generation was observed (Figure 3A). This effect was inhibited by LOS and DAL, as well by Tiron, a superoxide anion scavenger (Figure 3A). In the absence of DABK, a B1R agonist, ANG II at a high concentration (100 nM) increased superoxide anion generation when compared with vehicle. This effect was inhibited by LOS, but not by DAL, a B1R antagonist (Figure 3B).

### ANG II and B1R agonist have synergistic effects on ERK1/2 phosphorylation, protein synthesis and PCNA expression in VSMC

**Figure 4:**
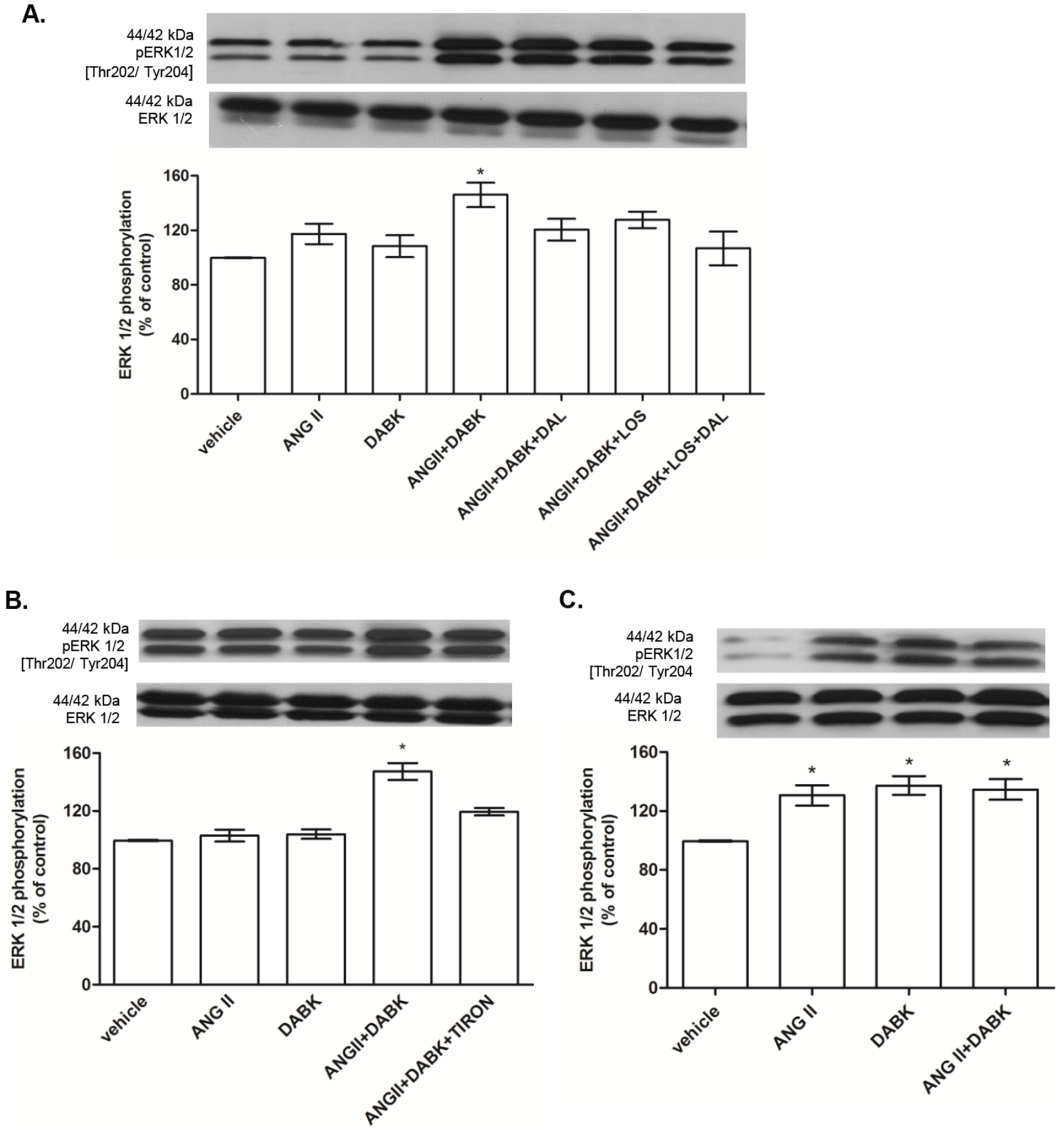
Effects of des-Arg^9^-bradykinin (DABK) and angiotensin II (ANG II) on ERK1/2 activation. [A] Aortic VSMC from Wistar rats treated with ANG II and/or DABK at low concentration (0.1 nM) in the presence of vehicle, losartan (LOS – 10 µM), des-arg9-leu8-bradykinin (DAL – 10 µM) and [B] TIRON (1 mM). Bar graph shows the ratio of phosphorylated/total ERK1/2 that was used as an indicator of ERK1/2 activity. Results are represented as mean±SEM of 5 experiments. *P<0.05 vs Vehicle. [C] Aortic VSMC from Wistar rats were treated with ANG II and/or DABK at high concentration (100 nM). Bar graph showed the ratio of phosphorylated/total ERK1/2 that was used as indicator of ERK1/2 activity. Results are expressed as mean ± SEM of 5 experiments. *P<0.05 vs vehicle.

ANG II and DABK at low concentration (0.1 nM) increased ERK1/2 phosphorylation (Figure 4A) only when added together and this effect was abolished by LOS, DAL and Tiron (Figure 4A and B). On the other hand, at a high concentration (100 nM) ANG II and DABK induced ERK1/2 phosphorylation when applied alone and no additional effect was observed when the peptides were applied simultaneously (Figure 4C).

**Figure 5:**
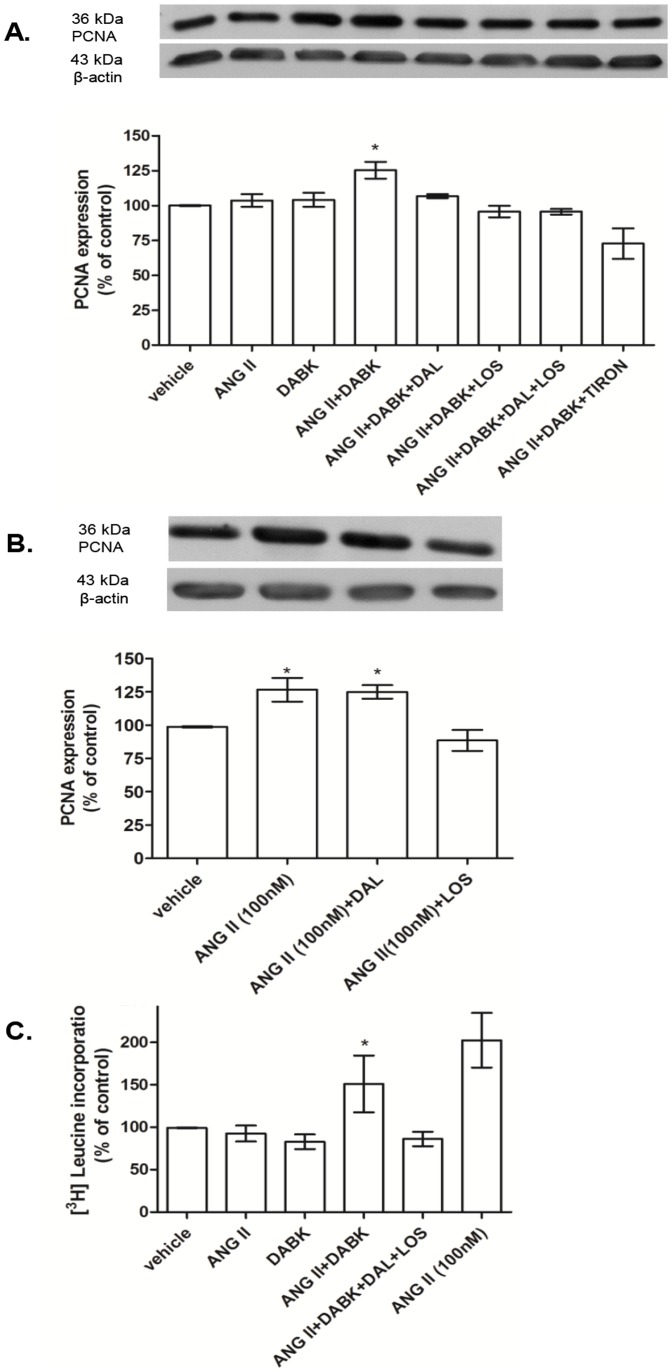
Effects of des-Arg^9^-bradykinin (DABK) and angiotensin II (ANG II) vascular smooth muscle cells (VSMC) growth. [A] Aortic VSMC from Wistar rats treated with ANG II and/or DABK at low concentration (0.1 nM) in the presence or not of losartan (LOS – 10 µM), des-arg9-leu8-bradykinin (DAL – 10 µM) and TIRON (1 mM). Bar graph shows PCNA expression normalized against the housekeeping protein β-actin. Results are represented as mean±SEM of 4 experiments. *P<0.05 vs Vehicle. [B] Aortic VSMC from Wistar rats with ANG II at high concentration (100 nM) in the presence or not of losartan (LOS – 10 µM), des-arg9-leu8-bradykinin (DAL – 10 µM). Bar graph shows PCNA expression normalized against the housekeeping protein β-actin. Results are expressed as mean±SEM of 5 experiments. *P<0.05 vs vehicle. [C] [H3] leucine incorporation in VSMC treated with ANG II and/or DABK at low concentration (0.1 nM) in the presence or not of losartan (LOS – 10 µM) and des-arg9-leu8-bradykinin (DAL – 10 µM) and VSMC treated with ANG II at high concentration (100 nM). Results are expressed as mean±SEM of 3 experiments and *P<0.05 vs vehicle.

PCNA expression and [H3] leucine incorporation were assessed as molecular markers of cell growth. As demonstrated in Figure 5A, ANG II and DABK at low concentration (0.1 nM), increased PCNA expression only when added together and these effects were abolished by LOS, DAL and Tiron. In the absence of DABK, ANG II at high concentration (100 nM) increased PCNA expression when compared with vehicle. This effect was not changed by DAL, a B1R antagonist, but was inhibited by LOS, AT1 receptor antagonist (Figure 5B). ANG II and DABK at low concentration (0.1 nM) increased [H3] leucine incorporation only when added together and LOS plus DAL abolished this effect. ANG II at high concentration (100 nM) increased [H3] leucine incorporation when compared with the control (vehicle) cells (Figure 5C).

## Discussion

Here we have demonstrated for the first time by in vivo and in vitro studies that B1R is upregulated in hypertensive ANG II-infused rats and contributes to vascular hypertrophy through a redox-sensitive ERK1/2 pathway.

We previously demonstrated that ANG II has a modulatory effect on B1R protein expression in the cardiovascular system [11], [12]. In addition, the increase in vascular B1R expression induced by ANG II relies on AT1 receptor activation, superoxide anion production by NADPH oxidase, PI3-kinase and NF-κB activation [11], [12], [21]. ERK1/2 plays a role in NF-κB activation by inflammatory stimulus that up regulates B1R [22]. However, ERK1/2 does not seem to be involved in ANG II-induced B1R up-regulation, since B1R antagonism reduced ERK1/2 activation but did not interfere with aortic B1R expression.

ANG II plays an important role in the etiology of cardiovascular diseases and also in the pathophysiology of cardiac and vascular hypertrophy [23]. A growing body of evidence suggests that ANG II effects on vascular structure are mediated by ROS production, through NADPH oxidase, and MAPK activation, which can mutually stimulate each other [24], [25]. In this study, we show that B1R activation, by endogenous DABK, contributes to vascular hypertrophy in ANG II-induced hypertension, by a mechanism involving ROS generation and ERK1/2 activation. In fact, it has been reported in isolated VSMC that DABK increases ERK1/2 activation via a cholera toxin–sensitive pathway [7]. In addition, kinins stimulate NADPH oxidase during inflammatory processes [26]. Furthermore, ANG II and DABK interaction increased PCNA expression and protein synthesis, supporting the hypothesis that DABK/ANG II interaction contributes to VSMC hypertrophy.

Whether the role of B1R expression in the cardiovascular system is beneficial or harmful remains inconclusive. After myocardial infarction [6] and in diabetic nephropathy [27] kinin receptors contribute to the protective effect of ACE inhibitors. On the other hand, it has also been demonstrated that blocking or deleting B1R protects the heart against injury and myocyte death [28], [29]. In agreement, we [30] and others [7] have demonstrated that B1R activation is related with several intracellular signaling pathways that have a key role in the development of vascular damage. Altogether, at present, there is no good explanation for the discrepancies described and investigations in different models of cardiovascular disease are needed to clarify these issues.

Deletion of kinin receptors gene causes downregulation of ANG II receptors [31]. Therefore, a decrease in vascular AT1R expression may be involved in the anti-hypertrophic effects of the B1R antagonist. However, our results do not support this hypothesis, since B1R antagonist infusion for 14 days did not interfere with AT1 receptor expression. It is important to note that, although AT1 receptor expression was not changed either by ANG II or B1R antagonist treatments, AT1 receptor activation is necessary for DABK/ANG II interaction, since AT1 antagonism in vivo prevented both the increased B1R expression and the vascular hypertrophy. LOS also prevented the synergism between low concentration of ANG II and DABK as well as the effects of ANG II at a high concentration, confirming the participation of AT1 receptors.

Synergistic interactions between kinins and ANG II receptors have also been described in pathological conditions, such as preeclampsia. In this condition, AT1 and kinin B2 receptors form stable heterodimers, resulting in enhanced activation of downstream signaling pathways via the AT1 receptor [32], [33]. Thus, it is might be possible that ANG II and DABK interact in VSMC through the formation of heterodimers between AT1 and B1Rs. However, more studies are needed to clarify this issue. Since the inhibition of vascular wall hypertrophy by B1R antagonism in vivo occurred in the absence of changes in systolic arterial blood pressure, we can conclude that AT1-B1R interaction is not involved in the hypertensive effect of ANG II. In fact, it is known that ANG II increases medial aortic VSMC via mechanisms that are independent of increases in systolic arterial blood pressure [4]. This is consistent with our data from VSMC, where ANG II and the B1R agonist increased PCNA expression and [H3] leucine incorporation, used as markers of cell growth. This finding led us to conclude that the effect of B1R antagonism on aorta hypertrophy is independent of changes on blood pressure levels.

The role of B1R on blood pressure control is still controversial. Whereas Martins et al. [34] reported that neither the B1R agonist DABK or the B1R antagonist DAL injected into the fourth ventricle modified blood pressure in spontaneously hypertensive rat (SHR) or Wistar–Kyoto rats, other studies showed an effect of B1R on blood pressure levels. In fact, centrally expressed B1R are involved in the maintenance of high blood pressure levels in SHR [35]. These authors showed that the B1R antagonist DAL injected into the lateral ventricle (i.c.v.) reduced blood pressure levels in SHR, but not in Wistar–Kyoto rat. Emanueli et al. [36] also reported that i.c.v. activation of B1R in SHR and Wistar–Kyoto rats evokes increases in blood pressure. Non-peptide kinin B1R antagonists, LF22-0542 and SSR240612, decreased systolic blood pressure in glucose-fed hypertensive rats [34], [37]. The authors suggested that the anti-hypertensive effect of these antagonists was most likely centrally mediated since a peptide B1R antagonist, which does not cross the blood–brain barrier, did not alter blood pressure [34], [37]. Since subcutaneous administration of the peptide antagonist DAL did not interfere with the development of hypertension, we suggest that peripherally expressed B1R might not be involved in ANG II-induced hypertension. Moreover, the physiological control of blood pressure does not appear to involve B1R, since B1R knockout mice do not display a hypertensive phenotype [38].

In summary, our data reveal a novel molecular mechanism involving ANG II and DABK in vascular cells that may amplify cellular responses. We show that ANG II and DABK, via AT1 and B1 receptors, synergistically activate the redox-regulated ERK1/2, which can regulate aortic VSMC growth in vivo and in vitro, contributing to vascular remodeling in hypertension. Our findings highlight an important cross-talk between the DABK and ANG II in the vascular system. Also they contribute to a better understanding of the mechanisms involved in hypertension-associated vascular remodeling.
